# SEOM-TTCC clinical guideline in nasopharynx cancer (2021)

**DOI:** 10.1007/s12094-022-02814-x

**Published:** 2022-03-18

**Authors:** Antonio Rueda Domínguez, Beatriz Cirauqui, Almudena García Castaño, Ruth Alvarez Cabellos, Alberto Carral Maseda, Beatriz Castelo Fernández, Leticia Iglesias Rey, Jordi Rubió-Casadevall, Virginia Arrazubi, Ricard Mesía

**Affiliations:** 1 Medical Oncology Intercenter Unit, Regional and Virgen de La Victoria University Hospitals, IBIMA, 29010 Málaga, Spain; 2 Medical Oncology Department. Catalan Institut of Oncology - Badalona. B-ARGO Group, IGTP, Badalona, Spain; 3 Medical Oncology Service, Hospital Marqués de Valdecilla, Santander, Spain; 4 Medical Oncolgy Department, Hospital Virgen de La Salud, Toledo, Spain; 5 Medical Oncology Service, Hospital Universitario Lucus Augusti, Lugo, Spain; 6 Medical Oncology Department, Hospital Universitario de La Paz, Madrid, Spain; 7 Medical Oncology Service, Complejo Hospitalario Universitario de Ourense, Ourense, Spain; 8 Medical Oncology Department, Hospital Josep Trueta. Catalan Institute of Oncology, Girona, Spain; 9 Medical Oncology Service, Complejo Hospitalario de Navarra. IdiSNA, Navarra Institute for Health Research, Pamplona, Spain

**Keywords:** Nasopharyngeal cancer, Clinical Practice Guidelines, Diagnosis, Treatment, Follow-up

## Abstract

Nasopharyngeal carcinoma (NPC) is distinct from other cancers of the head and neck in biology, epidemiology, histology, natural history, and response to treatment. Radiotherapy (RT) is the cornerstone of locoregional treatment of non-disseminated disease and the association of chemotherapy improves the rates of survival. In the case of metastatic disease stages, treatment requires platinum/gemcitabine-based chemotherapy and patients may achieve a long survival time.

## Introduction

Nasopharyngeal carcinoma (NPC) differs from other head and neck squamous cell carcinomas in epidemiology, histology, natural history, and response to treatment. Also displays a distinct racial and geographic distribution, which is reflective of its multifactorial etiology [1].

NPC is a rare disease in Europe, while is endemic in Southeast Asia.

Epstein-Barr virus (EBV) is, together with host genetics and environmental factors such as smoke, alcohol and wood dust-related occupations, among the well-known risk factors for these tumours.

There were 129 000 new cases of NPC reported in 2018, mainly in endemic areas while only about 5000 in Europe. In Spain, the world adjusted incidence rate for both sexes is 0.63 cases/100.000 inhabitants/year, so is an unusual tumor [2].

Age distribution differs in low-incidence areas compared with endemic areas. In low incidence areas, the incidence of NPC increases with age and has a bimodal peak: the first in adolescents and young adults and the second after 65 years of age, whereas in endemic areas, the incidence increases after 30 years of age, peaks at 40–59 years and decrease thereafter [3].

In Europe, during the period of 2000–2007, the 5-year survival rate for adults with NPC was 49% (www.rarecarenet.eu). Survival rates increased during 1999–2007 in Europe, except in Eastern Europe where it declined over time [3, 4]. The effect of age on survival is marked. Five-year survival rates were 72% in the youngest age group (15–45 years) and 36% in the oldest group of patients (65–74 years) [4].

The incidence of NPC is two- to threefold higher in males compared with in females. In general, the prognosis is better for women than men [1].

## Methodology

These Clinical Practice Guidelines have been produced by oncologists from the Spanish Group for the Treatment of Head and Neck Tumors (TTCC) and the Spanish Society for Medical Oncology (SEOM) with the objective to establish standard diagnostic and treatment guidelines that may be useful for clinical practice.

It includes the updated scientific evidence on in the diagnosis, treatment and follow-up of nasopharyngeal cancer, considering the indications approved in Spain. Recommended interventions are intended to correspond to the ‘standard’ approaches, according to current consensus among the experts that conceived and wrote the guidelines.

The relevant literature has been selected by the expert authors.

Levels of evidence and grades of recommendation have been applied using The Infectious Diseases Society of America grading system (Table 1) [5]. All the authors have contributed equally to the elaboration of these guidelines.

**Table 1:** Strength of recommendation and quality of evidence score

Category, grade	Definition
Strength of recommendation	
A	Good evidence to support a recommendation for use
B	Moderate evidence to support a recommendation for use
C	Poor evidence to support a recommendation
D	Moderate evidence to support a recommendation against use
E	Good evidence to support a recommendation against use
Quality of evidence	
I	Evidence from ≥ 1 properly randomized, controlled trial
II	Evidence from ≥ 1 well-designed clinical trial, without randomization; from cohort or case-controlled analytic studies (preferably from > 1 center); from multiple time series; or from dramatic results from uncontrolled experiments
III	Evidence from opinions of respected authorities, based on clinical experience, descriptive studies, or reports of expert committees

### Diagnosis

Definitive diagnosis is made by endoscopic-guided biopsy of the primary nasopharyngeal tumour [II, A]. In case of no clinical primary tumour visible at endoscopy, biopsy of nasopharyngeal tissue positive at magnetic resonance imaging (MRI) or positron emission tomography (PET) is suggested [3, 6].

Since the first sign of disease is often the appearance of neck nodes, it is frequent that patients undergo neck biopsy and/or neck nodal dissection. This procedure is not recommended since it may reduce the probability of cure and have an impact on late treatment sequelae. Nevertheless, if carried out (for example, if the primary tumour is not visible), node dissection without capsular effraction or ultrasonography-guided, transcutaneous tru-cut biopsy are the best options; node surgical biopsy should be avoided.

Determination of EBV on the histological sample by in situ hybridization (ISH) is indicated.

### Pathological diagnosis

The histological type should be classified according to the 4th edition of the World Health Organization (WHO) classification (Table 2) [7]. There are three pathological subtypes of nasopharyngeal carcinoma: keratinising squamous, non-keratinising, and basaloid squamous. Non-keratinising nasopharyngeal carcinoma can be divided into differentiated and undifferentiated tumours.

**Table 2:** WHO classification of nasopharyngeal carcinomas

Keratinizing squamous cell carcinoma	WHO type I
Non-keratinizing carcinoma	
Differentiated type	WHO type II
Undifferentiated type	WHO type III
Basaloid squamous cell carcinoma	

The keratinising subtype accounts for less than 20% of cases worldwide, and is relatively rare in endemic areas; the non-keratinising subtype constitutes most cases in endemic areas (> 95%) and is predominantly associated, even if not sufficiently causative, with EBV infection. Its role in keratinising cancer is less pronounced. EBV is identified by ISH by the presence of EBV-encoded RNAs in NPC tissue.

Basaloid squamous cell carcinoma was added to the WHO classification of head and neck tumors in 2005. There are few reported cases, but they are notable for an aggressive clinical course and poor survival.

The changing histology in the European populations suggests a modification of the natural history of the disease. The decreasing incidence of keratinizing nasopharyngeal cancer (WHO type I), which is smoke-related and at worse prognosis, may affect the over the time increase of survival [8].

We suggest obtaining pretreatment plasma Epstein-Barr virus (EBV) DNA levels as part of the diagnostic and staging evaluation. Pretreatment plasma EBV DNA levels are prognostic and have been associated with survival outcomes.

### Screening

In endemic regions, the use of plasma EBV DNA with a primer/probe assay targeting the BamHI-W region of the EBV genome, carried out in duplicate (at least 4 weeks apart) and coupled with endoscopic examination and MRI, showed a sensitivity and specificity in screening NPC of 97.1% and 98.6%, respectively [9]. The number of subjects needed to be screened to detect one case was 593. Its use can therefore only be recommended for detecting early asymptomatic NPC in endemic areas and is limited to those considered at higher risk (i.e., males aged 40–62 years) [III, A]. Although overall survival (OS) data for the screened population are not available, the 3-year progression-free survival (PFS) was significantly improved compared with a matched historical cohort [97% versus 70%; hazard ratio (HR) 0.10; 95% confidence interval (CI) 0.05–0.18] [9].

### Diagnostic evaluation and staging

The study should include [3]

1. A complete medical history and physical examination.
2. Full exploration of the head and neck area (including endoscopic examination and cranial nerves evaluation). A definitive diagnosis is made with endoscope-guided biopsy of the primary tumor. Incisional neck biopsy or nodal dissection should be avoided as this procedure will negatively impact subsequent treatment.
3. CT scan or MRI of the nasopharynx and base of the skull and neck (MRI preferred). When ordering an MRI, it is important to specifically request cranial nerve imaging, as a standard brain MRI does not provide adequate detail.
4. General blood count, and serum biochemistry, including liver function tests and alkaline phosphatase.
5. Tumour biopsy (EBER by ISH).
6. Baseline audiometric testing, dental examination, nutritional status evaluation, ophthalmological and endocrine evaluation.
7. Plasma EBV DNA. The addition of pretreatment plasma EBV DNA to the 8th edition of the American Joint Committee on Cancer (AJCC) tumor, node, metastasis (TNM) staging system has improved its prognostic performance.
8. 18F-FDG-PET/CT is indicated for patients with advanced nodal disease (stage N3), clinical evidence suggesting distant metastases, or an EBV DNA load ≥ 4000 copies/mL, since these patients are at high risk for distant metastases. Metastatic lymph nodes and bone lesions are better detected by 18F-FDG-PET/CT.

Levels of evidence and grades of recommendation applied for recommendations for diagnostic and staging evaluation are shown in Table 3.

**Table 3:** Recommendations for diagnostic and staging evaluation

Diagnostic and Staging	Level of evidence
Definitive diagnosis is made by endoscopic-guided biopsy of the primary nasopharyngeal tumour; diagnostic neck biopsy and/or neck nodal dissection should be avoided	[II, A]
Determination of EBV on the histological specimen by ISH is indicated	[III, B]
Analysis of EBV DNA in plasma is useful for screening at-risk populations for nasopharyngeal carcinoma. It can detect the cancer at an early stage with a superior treatment outcome compared with the unscreened population	[III, A]
For the initial diagnostic evaluation of nasopharyngeal carcinoma, we suggest endoscopically guided biopsy of the primary tumor and magnetic resonance imaging (MRI) of the nasopharynx, skull base, and neck to assess locoregional disease extent	[III, B]
For patients with advanced nodal stage (N3) or clinical or biochemical evidence of distant metastases, we offer additional imaging with positron emission tomography (PET) or integrated PET/computed tomography (CT) imaging if available. Otherwise, bone scan and CT of the chest and abdomen may be obtained	[III, B]
We suggest obtaining pretreatment plasma EBV DNA levels for their prognostic significance. There is emerging evidence supporting serial measurement of plasma EBV DNA levels to assess treatment response or monitor for recurrence	[III, B]

NPC is clinically staged according to the American Joint Committee on Cancer (AJCC) staging classification 8th edition [10] (Table 4). This staging system provides important prognostic information and guidance for choosing the appropriate treatment for patients with nasopharyngeal carcinoma (Table 5).

**Table 4:** Nasopharyngeal cancer TNM staging AJCC UICC 8th edition

Primary tumor (T)	
T category	T criterio
TX	Primary tumor cannot be assessed
T0	No tumor identified, but EBV-positive cervical node(s) involvement
Tis	Tumor in situ
T1	Tumor confined to nasopharynx, or extension to oropharynx and/or nasal cavity without parapharyngeal involvement
T2	Tumor with extension to parapharyngeal space, and/or adjacent soft tissue involvement (medial pterygoid, lateral pterygoid, prevertebreal muscles)
T3	Tumor with infiltration of bony structures at skull base, cervical vertebra, pterygoid structures, and/or paranasal sinuses
T4	Tumor with intracranial extension, involvement of cranial nerves, hypopharynx, orbit, parotid gland, and/or extensive soft tissue infiltration beyond the lateral surface of the lateral pterygoid muscle

Regional lymph nodes (N)	
N category	N criterio
NX	Regional lymph nodes cannot be assessed
N0	No regional lymph node metastasis
N1	Unilateral metastasis in cervical lymph node(s) and/or unilateral or bilateral metastasis in retropharyngeal lymph node(s), 6 cm or smaller in greatest dimension, above the caudal border of cricoid cartilage
N2	Bilateral metastasis in cervical lymph node(s), 6 cm or smaller in greatest dimension, above the caudal border of cricoid cartilage
N3	Unilateral or bilateral metastasis in cervical lymph node(s), larger than 6 cm in greatest dimension, and/or extension below the caudal border of cricoid cartilage

Distant metastasis (M)	
M category	M criterio
M0	No distant metastasis
M1	Distant metastasis

*TNM* tumor, node, metastasis; *AJCC* American Joint Committee on Cancer; *UICC* Union for International Cancer Control

**Table 5:** Prognostic stage groups

Stage 0	Tis	N0	M0
Stage I	T1	N0	M0
Stage II	T1, T2	N0,N1	MO
Stage III	T0, T1, T2, T3	N2, N0,N1, N2	M0, M0
Stage IVA	T4, Any T	N0,N1, N2, N3	M0, M0
Stage IVB	Any T	Any N	M1

### Treatment

The optimal treatment strategy for patients with NPC should be discussed within a multidisciplinary team (MDT). Treatment of patients in high-volume facilities is recommended.

### Management of local/locoregional disease

Radiotherapy (RT) is the cornerstone of locoregional treatment for NPC. IMRT has shown the best results with fewer late effects than conventional RT, with a 5-year disease-specific survival rate of 92–94% [11–14]. In addition to the primary tumor and pathological nodes, both sides of the neck (levels II-V) and the retropharyngeal nodes should be included because of the high incidence of occult neck node involvement [15]. The dose should be 70 Gy in 33–35 fractions (2.0–2.12 Gy per fraction) delivered over 7 weeks (once daily, five fractions per week) in the primary tumor and affected lymph node areas and 50–60 Gy for the treatment of potential at-risk sites [IA]. Both sequential boost and simultaneous integrated boost radiotherapy may be offered [16] [II, B].

### Early stages (I and II)

The treatment for stage I and II tumors is RT alone, except for some special situations that suggest increased risk of recurrence.

The administration of concomitant chemotherapy with RT in stage II patients remains poorly defined. The use of CRT with CDDP 30 mg/m^2^ weekly + conventional RT showed benefit in overall survival (OS), progression-free survival (PFS) and distant metastases-free survival (DMFS) but not in locoregional relapse-free survival (LRFS) [17] [II, B]. There were no statistically significant differences when the RT used was IMRT [18, 19]*.* Because this stage consists of subgroups with different risk of distant metastases, the use of chemotherapy may be considered in N1, bulky T2 or high EBV DNA level patients (> 4000 copies per mL) [II, B].

### Locally advanced stages (III and IV A/B)

#### Concurrent chemoradiotherapy (CRT)

Concurrent CRT with cisplatin 100 mg/m^2^ every 3 weeks is the standard treatment for locoregionally advanced NPC carcinoma and substantially improves overall survival, locoregional and distant control compared to exclusive RT [20, 21] [I, A].

Weekly cisplatin [22,23] and carboplatin [21] can be considered if standard treatment is contraindicated [II A, II B].

Since distant metastases are the main cause of relapse and death despite this treatment, different strategies have been developed to improve these results.

### Induction chemotherapy

Different randomized trials [24–26] and meta-analyses [27–32] have shown that induction chemotherapy (IC) carries a benefit in distant relapse-free survival compared to standard chemoradiotherapy (CRT) in patients with locally advanced nasopharyngeal carcinoma (NPC) at high risk of developing metastases (non-keratinising histology, significant lymph node involvement, very fast-growing tumors), and, as a consequence, in overall survival (OS) [I, A]. Most of them used platin and 5-fluorouracil (PF) or docetaxel, cisplatin and 5-fluorouracil (TPF) and patients with T3-4N0 disease were excluded.

It is important to take into account the characteristics of the patient and the toxicity associated with IC when selecting this strategy, so that it does not compromise compliance with CRT. IC with cisplatin and gemcitabine (CG) followed by CRT has got greater efficacy than CRT alone in terms of recurrence-free survival (RFS), distant recurrence-free survival (DRFS) and OS with greater acute hematologic and gastrointestinal toxicity, but without increasing late toxicity and with acceptable compliance of the treatment [33] [I, A].

Although there is no face-to-face comparison between IC-CRT and CRT-AC, results from different analyses suggest that IC achieves superior efficacy results due to better distance disease control [21, 34, 35].

### Adjuvant chemotherapy

The addition of adjuvant chemotherapy (AC) to CRT remains controversial [II, B].

Intergroup-0099 trial established CRT followed by AC with PF as the standard treatment of locoregionally advanced (stage III–IVA) NPC, given the superior overall survival over conventional RT alone [36].

With the implementation of IMRT in clinical practice, most of subsequent randomized studies and meta-analyses have failed to demonstrate an OS benefit of AC when added to CRT [37–39], even a trial that randomized patients to receive AC with CG based on detectable plasma EBV DNA after CRT [40].

In addition to this, it is critical an accurate selection of patients due to toxicity and compliance of AC after CRT [41].

Recently, two Asian trials have been reported that obtain a benefit in recurrence-free survival (RFS) with adjuvant capecitabine after CRT in high-risk locally advanced NPC [42, 43].

### Recommendations for locoregional treatment

**Fig. 1:**
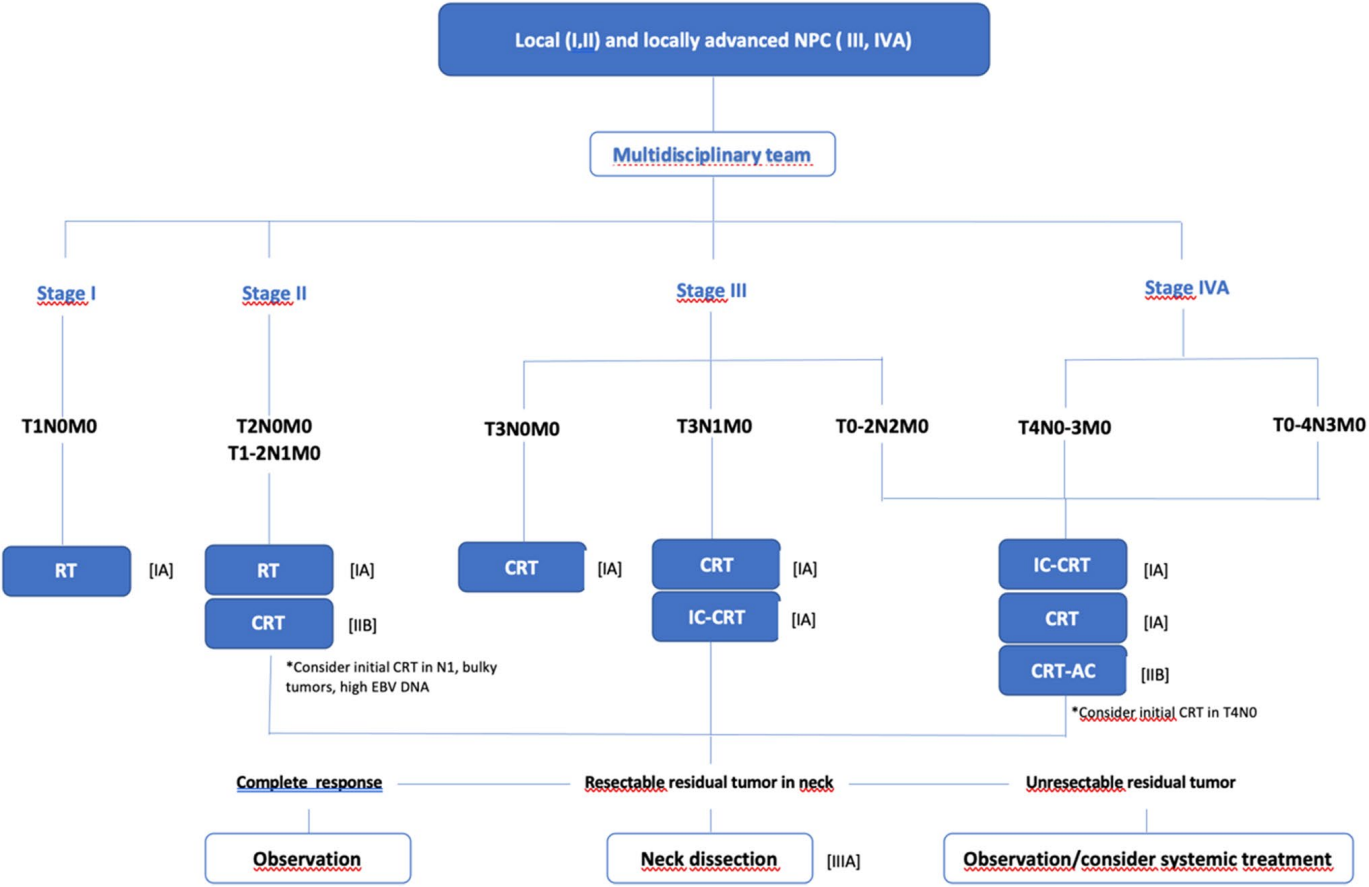
Treatment options in early and locally advanced NPC. Radiotherapy (RT): mandatory IMRT. Chemotherapy schemes: CRT: cisplatin 100 mg/m^2^ every 3 weeks (*preferred);* weekly cisplatin; carboplatin. IC: cisplatin and gemcitabine (CG) (*preferred);* docetaxel, cisplatin and 5-fluorouracil (TPF), cisplatin and docetaxel (TP); cisplatin and 5-fluorouracil (PF). C: cisplatin and 5-fluorouracil (PF)

Treatment options (with strength of recommendation and quality of evidence) for early (stages I and II) and locally advanced (stages III–IVA) NPC are shown in Fig. 1.

### Management of locoregional recurrences and metastatic disease

Locoregional recurrences may be curable, so a local treatment is often recommended [II, B], albeit expecting significant sequeale. The main therapeutic options are surgery (open or endoscopic nasopharyngectomy [44]) and/or re-irradiation (IMRT, brachytherapy, radiosurgery (SRS) and stereotactic-body RT (SBRT) with or without concurrent chemotherapy [45].

There is no strong evidence to choose between reirradiation and surgery [46]. Recurrence volume, disease-free interval, performance status and comorbidities [45–47] are important decision factors. For the evaluation of surgery, referral of the patient to a high-volume center would be desirable.

Prognostic factors for surgery are rT and rN stage [48], surgical approach, feasibility of adjuvant re-irradiation [49] and pre-treatment circulating EBV DNA [50] (as on more advanced disease). Lymphatic recurrences in the neck can be treated with radical or selective neck dissection [III, A]. rT1-3 tumors might benefit more from endoscopic nasopharyngectomy than from IMRT [51]. Nasopharyngectomy is contraindicated in presence of carotid invasion or intracraneal invasion [II, E].

Patient selection for re-irradiation is essential due to very high incidence of complications [III, B]. Prognostic factors for re-irradiation are: recurrence volume, age, prior RT toxicity, advanced rT stage and higher re-IMRT dose [52]. Preliminary results with proton and carbon ion therapy are promising.

Palliative chemotherapy with cisplatin and gemcitabine is the standard of care in non-curable relapses and metastatic NPC. A phase III trial [53] achieved an improvement in outcome with cisplatin and gemcitabine compared to cisplatin and 5 fluorouracil once every 3 weeks. 362 Asian patients were included and most of them (83%) had a non-keratinising undifferentiated NPC (type III). The median progression free survival (PFS) was 7 months (4.4–10.9) in the experimental arm and 5.6 months (3–7) in the standard arm (HR 0.55; 95% CI 0·44–0·68, *p* < 0·0001) achieving the main objective of the study. The median overall survival (OS) was also better in the experimental arm (29.1 months [12–31.5] vs 20.9 months [0.45–0.84]). The response rate was higher in the cisplatin-gemcitabine group (64% vs 42%). Different treatment-related grade 3–4 adverse events were described with more haematologic toxicity in the gemcitabine arm and more mucositis in the fluorouracil arm [I, A]. For patients with contraindication to either of the two drugs, cisplatin/carboplatin + 5-fluoruracil can be indicated.

In patients with newly diagnosed metastatic NPC and who had achieved a response with fist line chemotherapy, the addition of locoregional radiotherapy improves OS. A phase III trial [54] randomized 126 patients to receive either chemotherapy or locoregional intensity-modulated radiotherapy (IMRT) 70 Gy plus chemotherapy. The 24 months OS was 76.4% in the combination arm versus 54.5% in the chemotherapy alone arm (HR 0.42; 95% CI 0.23–0.57). Non-significant differences in toxicity were observed [II, A].

Immunotherapy with immune-checkpoint inhibitors (specifically toripalimab and camrelizumab) combined with chemotherapy has shown an improvement in PFS in preliminary results reported in Asian population [55, 56]. Final results are awaited.

No standard second line has been stablished. Some agents have proved activity (paclitaxel, docetaxel, 5-FU, capecitabine, irinotecan, vinorelbine, ifosfamide, doxorubicin, oxaliplatin and cetuximab) [III, B]. Immunotherapy with immune-checkpoint inhibitors has shown promising activity too [57–59] [III, B]. Nivolumab, pembrolizumab and camrelizumab showed 20–34% of response rate in different phase I/II trials. Phase III trials are ongoing to elucidate the role of immunotherapy.

Oligometastatic patients may benefit from local treatment of the metastatic sites after a favourable course with chemotherapy [60] [II, B].

**Fig. 2:**
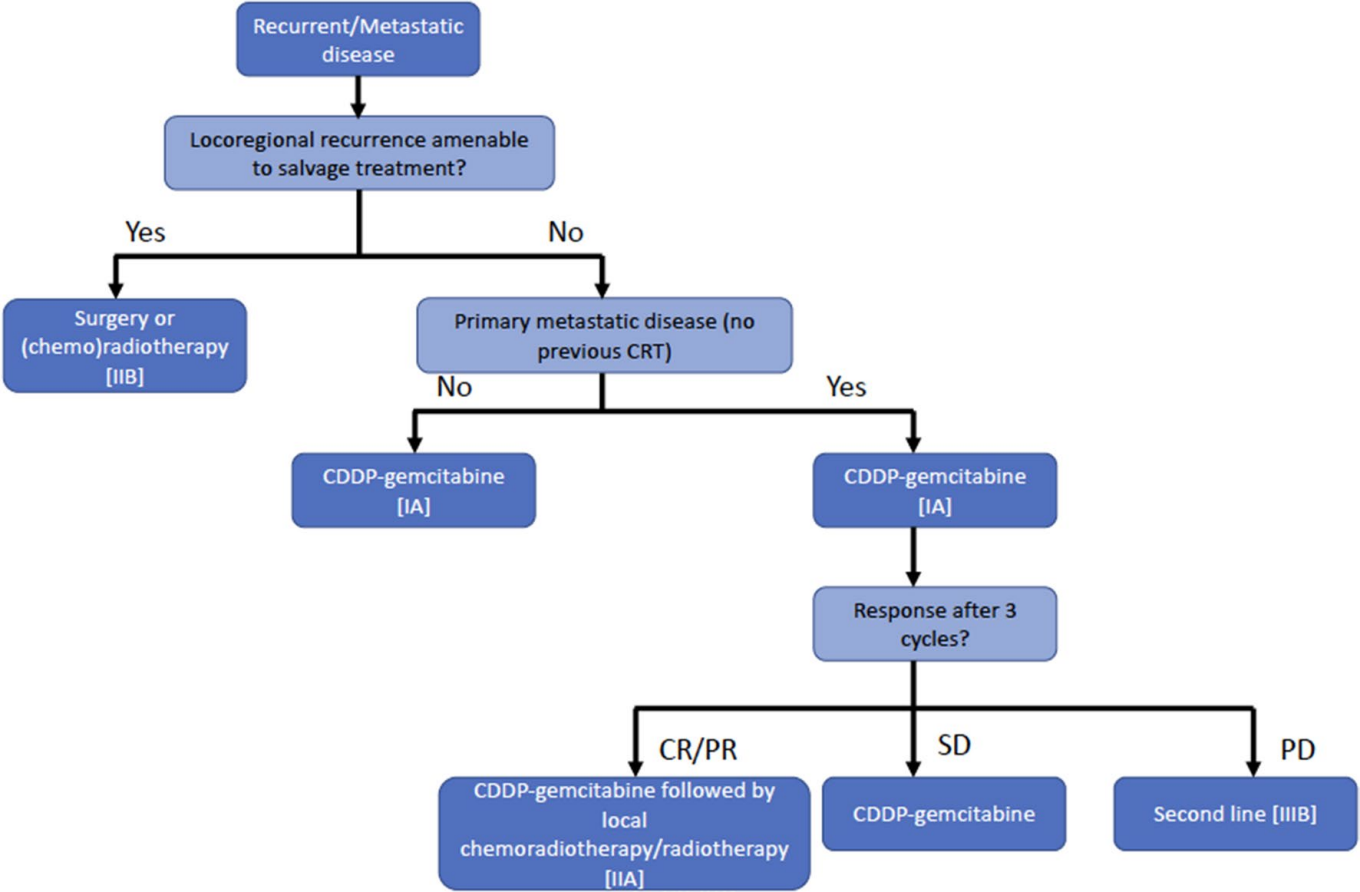
Treatment options in locoregional recurrences and metastatic disease. *CR*: complete response. *PR*: partial response. *SD*: stable disease. *PD*: progressive disease

Recommendations for locoregional recurrences and metastatic disease treatment are shown in Fig. 2.

### Follow-up, long-term implications and survivorship

Evaluation of response in the nasopharynx and neck with clinical and endoscopic examination and imaging studies should be performed. The first radiological imaging is suggested 3 months after treatment completion. Sensitivity of MRI and metabolic imaging (i.e., PET) are similar [II, B], whereas the specificity of PET is higher and so helps to differentiate between post-irradiation changes and recurrent tumours [61].

Further follow-up for patients includes periodic (every 3 months in the first year, every 6 months in the second and third year and annually thereafter for the first 5 years) examination of the nasopharynx (with endoscopic) and neck, cranial nerve function and evaluation of systemic complaints to identify distant metastasis [V, B] (Table 6). For T2-T4 tumours, MRI might be used on a 6 monthly basis to evaluate the nasopharynx and the base of the skull at least for the first few years after treatment [V, B].

**Table 6:** Follow-up recommendations for nasopharyngeal carcinoma

Final assessment (2–3 months after the end of treatment)	*Local and regional exam plus nasopharyngeal fibroscopy *FDG-PET/CT and/or RMI [II, B]
First year	*Local and regional exam plus nasopharyngeal fibroscopy (every 3 to 4 months) *CT/MRI (every 6 months) *Chest X-ray, thyroid function test (yearly) [V, B]
2–3 years	*Local and regional exam plus nasopharyngeal fibroscopy (every 6 months) *CT/MRI (every 6 months) *Chest X-ray, thyroid function test (yearly) [V, B]
4–5 years	*Local and regional exam plus nasopharyngeal fibroscopy (every 6 months) *Chest X-ray, thyroid function test, CT/MRI (yearly) [V, B]

Plasma EBV DNA is a promising marker for the diagnosis of recurrence [II, B] and should be evaluated at least every year [62] [V, B].

Evaluation of thyroid function in patients who have received RT to the neck is recommended annually and thoracic imaging test should be carried out at least once a year [V, B].

Patients should be followed to diagnose late toxicities, paying special attention to the recognition of late treatment-related toxicities, mainly consisting of xerostomia, trismus, hearing impairment, temporal lobe necrosis (TLN), cognitive impairment, cranial nerve injuries. The employment of IMRT instead of 2D-RT has substantially reduced these late events with the exception of TLN. The risk is higher depending on the stage, the addition of chemotherapy and the total dose of radiation therapy to the temporal lobe [63, 64].

Patients with nasopharyngeal cancer have a lower risk of second neoplasms than other tobacco-related head and neck sites [65, 66]. Therefore, long-term follow-up should not focus on the detection of second neoplasms [III, B]. Even so, the risk of bone cancer in irradiated areas must be taken into account [67].
